# Evaluating a Dyadic Intervention on Risk Reduction Among People Who Inject Drugs

**DOI:** 10.1177/1178221818799753

**Published:** 2018-09-10

**Authors:** Natalie Flath, Karin Tobin, Aleks Mihailovic, Paige Hammond, Carl Latkin

**Affiliations:** 1 Department of Health, Behavior and Society, Johns Hopkins Bloomberg School of Public Health, Baltimore, MD, USA; 2 Wilmer Eye Institute, Johns Hopkins School of Medicine, Baltimore, MD, USA

**Keywords:** Harm reduction, health behavior, social networks

## Abstract

Among 100 people who inject drugs enrolled in a peer mentorship intervention aiming to promote injection-related risk reduction behavior change, we evaluated the role of participation in a dyad session on reducing sharing of syringes and cookers in the past 6 months. Dyad participants (n = 69) invited an injection, sex partner, or family member to the study site to reinforce learnt behavior change tools by practicing communication skills and risk reduction lessons. In all, 31 participants did not participate in the dyad session. We descriptively assessed changes in sharing injection equipment between the 2 time points of pre- and postintervention using the tests of proportions by dyad participation. Multivariable logistic regression adjusted for sex was used with an interaction term (time points × dyad participation) to evaluate the dyad effect. Dyad participants reported reduced syringe and cooker sharing at postintervention (sharing syringe: 17% versus 39%, *P* < .05 and cooker: 32% versus 59%, *P* < .01). There was no difference between the dyad group’s sharing injection equipment behavior after the intervention (sharing syringes: adjusted odds ratio [aOR] 0.76; 95% confidence interval [CI] 0.1-3.9 and cookers: aOR 0.72; 95% CI 0.1-3.5). The role of the dyad session alone on risk taking was not effective. With a small sample size, it is important to continue to evaluate the nature of peer-based dyadic experiences in future studies.

## Introduction

Injection drug use remains a key transmission mode for new human immunodeficiency virus (HIV) and hepatitis C (HCV) cases in the United States.^1–3^ Despite the dissemination about risks associated with illicit drug injection, greater availability of sterile injection equipment, and decreased rates of sharing behaviors, sharing injection equipment continues to occur (ie, syringes and cookers: containers used to prepare a drug via mixing or heating).^4^

Existing research has indicated that closeness and intimacy between injection partners are related to and may be responsible for elevated injection risk.^5–8^ Drug procurement, preparation, and injection are often social activities, between two or more other individuals. These interactions can vary from longstanding partnerships to anonymous interactions.^9^ Female injecting partners who are often reliant on partners for drug procurement are subsequent utilizers of shared injection, heightening their risk.^5,10^ The division of drug preparation responsibility influences sharing behavior dynamics among both heterosexual intimate and familial partners.^5,7,8^ These relationship dynamics pose both challenges and opportunities in the development and promotion of targeted behavioral change interventions.

Considering the clustering of risk behaviors in injection relationships, dyadic interventions may be an effective way to promote behavioral change techniques for HIV and HCV prevention. Dyadic interventions have demonstrated improvement of health behaviors in a variety of settings such as those targeting tobacco, alcohol and drug misuse, and HIV prevention and care.^11–14^ Several randomized trials have recently explored the effects of couples-based interventions on reducing HIV-related injection risk among intimate couples. McMahon et al^6^ reported that a couples-based counseling and testing (CBCT) intervention aimed at reducing injection and sexual risk behaviors among women who use illicit drugs (crack, cocaine, and heroin) was more effective than CBCT alone. Similarly, 2 related couples-based HIV/STI (sexually transmitted infection) risk reduction interventions conducted among risky intimate heterosexual partners found that compared with individual or wellness only promotion, the couples risk reduction intervention more significantly reduced sexual- and drug-related risk behavior.^5,15^ Beyond these few studies, literature on dyadic interventions for behavior change within peer partnerships remains relatively sparse and mainly focuses on sexual risk reduction among intimate couples.

Given the limited exploration of dyadic interventions on injection-related risk among intimate and nonintimate couples (ie, type of dyads), we examine the effect of participating in an optional dyad session on sharing injection equipment among participants who were enrolled in a 7-session peer-based HIV risk reduction behavioral change intervention.^16^ The dyad session was designed to allow the participant time and space to practice and teach the learnt risk reduction skills to an injection, sexual risk partner, or family member selected from their social network. A dyadic component is intended to increase self-efficacy and reinforce behavior change. Self-efficacy is a key mediating factor of behavior change and may be an important concept to integrate within behavioral change interventions to reduce injection-related risk behavior.^17,18^ We hypothesize that those who participated in the dyad session will report less syringe and cooker sharing at follow-up compared with those who did not participate.

## Methods

### Study population

Data for this study are derived from the STEP into Action study, an evaluation of a 7-session HIV prevention and Harm Reduction Peer-Based training intervention.^16^ Details on the recruitment and enrollment procedures can be found in the work by Tobin et al. In brief, participants were recruited using a variety of methods including word of mouth, street-based outreach, and referrals from community partners from April 2005 to December 2009 in urban, high drug-trafficked settings in Baltimore City, Maryland. At this time, needle syringe programs were continuously and widely available.^19^ To the author’s knowledge, no secular trends occurred during the study period. Inclusion criteria were as follows: aged 18 and older, self-report injection in the previous 6 months, and willing to talk with peers about risk reduction. Enrolled participants, called index participants, completed a baseline risk assessment and were randomized into the experimental arm or an equal attention control condition. The experimental arm included 7 sessions (5 group-based, 1 individual-based, and 1 optional dyad session) that focused on training index participants with communication skills to promote risk reduction with individuals in their social network. The dyad session was promoted to allow the time and space for the index participant to teach the learnt curriculum to one of their preselected network members who were not participating in the intervention. Specifically, index dyad participants promoted risk reduction techniques associated with syringe and cooker sharing to their network member. The session was intended for the index participant to practice and role model injection risk reduction techniques and to goal-set. For the purpose of this analysis, only index dyad participants enrolled in the intervention were included. Index dyad participants were interviewed prior to the intervention and 6 months after the completion of the sessions. The interviews were collected face to face until sensitive questions arose (ie, sexual and drug use behavior), which lead to an audio-assisted self-administered questionnaire.

Network members accompanying the indexes to the session were identified using an ego-centric social network inventory and delineated during the baseline visit. The index’s network partners were peers with various risk relationships. In all, 44 dyad partners were both injection drug use and sexual partners, 10 were injection drug use only partners, 14 were sexual only partners, and 6 were neither. Six dyad network partners were family members. The dyad session was a 90-minute structured session that was cofacilitated by a male and female peer-trained advocate. Written and oral consents were provided to the participants prior to study initiation. The study was approved by the Institutional Review Board at Johns Hopkins Bloomberg School of Public Health.

### Measures

Outcomes: To assess sharing syringes we asked the question, “In the past six months, when you injected drugs, how often did you use a needle or tool that you were not sure was clean?” The question, “In the past six months, when you injected drugs, how often did you use a cooker that had been used by another person?” was used to assess sharing cookers. The outcome was dichotomized as yes if shared at least once within the past 6 months and no if reported 0.

Dyad session participation: We compared index participants who attended the dyad session with a network partner to those indexes who did not participate at all.

Independent variables: Age (dichotomized at the median of 45 years old for analysis purposes), race (African American versus white and other), sex, education level (less than high school versus high school or more), homelessness in the past 6 months (homeless versus not homeless), employment status, frequency of injecting heroin, HIV status derived from a positive reaction to the OraSure antibody test result among 89 participants who completed the test, and report of needle exchange participation in the past 6 months among 68 participants. Frequency of injecting heroin was categorized as injecting at “least once daily” (i.e., almost everyday, everyday, 2-5 times daily, greater than 5 times per day), “at least once per week” (i.e., 1-2 times per week, 3-4 times per week), and “less than weekly” (i.e., less than once per week).

### Analysis

To examine differences by dyad participation, demographic and injection-related characteristics were compared using Fisher exact tests among 100 index participants who were enrolled in the 7-session intervention. In all, 14 people were excluded from the analysis because they were lost to follow-up or did not report recent injecting at postintervention. No demographic, harm reduction service utilization, or equipment sharing differences were found between those included versus excluded. The change in proportion by level of dyad participation comparing pre- and postintervention was evaluated to visualize a preliminary effect of dyad participation on sharing syringes and cookers. The change in proportions was evaluated using the tests of proportions (prtest).To identify whether dyad participation was independently associated with sharing syringes and cookers at each time point (preintervention and postintervention), multivariable 2 logistic regression models were conducted for each outcome controlled for sex. Sex and needle exchange utilization differed between dyad participation and were considered for model inclusion. Needle syringe exchange did not change the model estimates with inclusion and as nearly one-third of the responses for needle exchange utilization were missing, we excluded to hold the models parsimonious. We imputed needle syringe utilization nonresponses to 0 and found no bivariable statistical difference with dyad participation. Two final models were conducted to assess the effect of dyad participation on both sharing behaviors at postintervention compared with baseline with an interaction term (time points × dyad participation). Stata version 11.0 was used to perform analyses.^20^

## Results

Table 1 illustrates baseline characteristics of the participants by dyad participation. Most of the 100 participants were older than 45 years old and African American. More than two-thirds (41%) had reported sharing syringes and more than half shared cookers (59%). Those who participated in the dyad session (n = 69) were more likely to be female and have attended a needle exchange (*P* < .05).

**Table 1.** Baseline characteristics by index participation in a dyadic component of an HIV risk reduction intervention among people who inject drugs who were followed-up postintervention (n = 100).

	Total	Dyad participation	No-dyad participation
	n 100	No. (%) 69	No. (%) 31
*Socio-demographic*			
Age >45 y (mean)	55	39 (57)	16 (52)
Race			
African American	85	61 (88)	24 (77)
White and other	15	8 (12)	7 (23)
Sex*			
Male	55	30 (43)	25 (81)
Female	45	39 (57)	6 (19)
Education level			
High school or more	40	27 (39)	13 (42)
Less than high school	60	42 (61)	18 (58)
Homeless <6 mo	37	25 (36)	12 (39)
Unemployed <6 mo	93	63 (91)	30 (97)
*Heroin injection use frequency (n = 92)*			
>Daily	57	37 (58)	15 (54)
At least once per week	32	22 (34)	7 (25)
Less than weekly	12	5 (8)	6 (21)
*HIV positive status (n* = *89)*	12	7 (11)	4 (16)
*Needle exchange attendee (n* = *68)*	63	32 (71)	11 (48)
*Sharing syringes*	41	27 (39)	14 (45)
*Sharing cookers*	59	41 (59)	18 (58)
*P > .05			

Abbreviation: HIV, human immunodeficiency virus.

Table 2 demonstrates proportional change of recent syringe and cooker sharing by dyad participation. Reductions in sharing syringes and cookers were observed for both dyad and nondyad participants from baseline to 6-month follow-up. A statistically significant lower proportion of sharing syringes and cookers were reported at postintervention compared with preintervention among those who participated in the dyad session (syringe sharing: 17% versus 39%, *P* < .05 and cooker sharing: 32% versus 59%, *P* < .05). Likewise, the proportion of sharing syringes and cookers at postintervention among nondyad participants compared with preintervention were lower but not significantly different (syringe sharing: 26% versus 45% and cooker sharing: 35% versus 58%). The differences in proportions between pre- and postintervention were greater among the dyads compared with the nondyads for both outcomes.

**Table 2.** Comparing the proportional change in recent syringe and cooker sharing between pre- and postintervention by index dyad participation (n = 100).

Intervention	n	HIV risk factor	Preintervention	Postintervention	Change in proportions %
			%	%	
		*Shared syringes*			
Dyad^a^	69		39	17	−22
Nondyad	31		45	26	−19
		*Shared cookers*			
Dyad^a^	69		59	32	−27
Nondyad	31		58	35	−23

Abbreviation: HIV, human immunodeficiency virus.

a Significantly different sharing outcomes between pre- and postintervention using the tests of proportions (prtest) at the *P* < .01 level.

### Multivariable regression

There was no significant difference in syringe and cooker sharing outcomes by dyad participation preintervention after adjusting for sex and needle exchange utilization (Table 3). At postintervention, there was no significant difference; however, the effect was descriptively reduced (sharing syringes: adjusted odds ratio [aOR] 0.77; 95% confidence interval [CI] 0.3-2.3 and cookers: aOR 0.76; 95% CI 0.3-2.0).

**Table 3.** Multivariable results on the effect of index dyad participation on sharing syringes and cookers at pre- and postintervention among 100 people who inject drugs.

	Shared syringes	Shared cookers
	aOR (95% CI)^a^	aOR (95% CI)^a^
	Preintervention	
Dyad versus nondyad	1.0 (0.4–2.6)	1.0 (0.4–2.6)
	Postintervention	
Dyad versus nondyad	0.77 (0.3–2.3)	0.76 (0.3–2.0)

Abbreviations: aOR, adjusted odds ratio; CI, confidence interval.

a aOR, adjusted for sex. (Did not display the null results of an interaction effect [pre-post intervention and dyad participation]).

When interacting the time points of pre- and postintervention outcomes and dyad participation, there was no effect. However, the direction of the point estimate aligns with the proposed hypothesis. After the intervention, compared with preintervention, there was no effect of sharing syringes and cookers among dyad participants compared with those who did not participate, although the direction of the odds ratio shows a reduction (sharing syringes: aOR 0.76; 95% CI 0.1-3.9 and cookers: aOR 0.72; 95% CI 0.1-3.5).

## Conclusions

We sought to evaluate the role of an optional peer-based dyadic session on the reduction in sharing injection-related equipment among people who inject drugs. We found that the role of the dyad session alone on risk taking was not effective. There was no significant difference in injection related HIV risk behavior by dyad participation; however, when stratified, it was less common for dyad participants to share syringes and cookers after the intervention compared with nondyad participants. It is important to note that dyad participants prior to the intervention were less commonly reporting syringe sharing compared with nondyads. With a small sample, the dyadic effect may have been missed. It is important to further evaluate the nature of a peer-based dyadic experience meant to reinforce learnt skills in future studies. A larger scale experimental study is warranted to affirm the hypothesis.

These results do not support the existing literature incorporating concepts of dyadic relationships in HIV prevention interventions.^11–14^ A number of randomized trials have reported the effectiveness of couples-based interventions on reducing injection-related risks.^5,15,21^ El-Bassel et al^5^ demonstrated the modality effect of an HIV/STI risk reduction couples-based randomized intervention on reducing the proportion of sexual risk acts among intimate injecting partners who were sociodemographically representative to our study population. Furthermore, Gilbert et al^15^ observed that a greater decrease in the proportion of risky injection acts among intimate injecting partners in Kazakhstan who participated in a couples-based HIV/STI risk reduction intervention in comparison with couples who participated solely in individually oriented sessions.

Previous studies have studied injection-related risk outcomes as composite measures. To our knowledge, we are the first to present findings of a dyadic effect on cooker sharing alone. We observed a decrease in the proportion of cooker sharing among dyad participants only, despite not observing a statistically significant independent effect. The STEP into Action intervention focused training on safe-drug splitting techniques.^16^ This was a common theme during the dyad session and was reinforced by the participants. El-Bassel et al^5^ included cooker sharing in a composite measure describing sharing injection equipment (ie, shared works, cooker, cotton, or rinse water) to demonstrate the effectiveness of a couples risk reduction intervention. They reported a decrease in the proportion of injection risk by participation in the couple risk reduction intervention, although not statistically significant. Specialized techniques driven to reduce injection-related risk and corresponding outcomes may be an important component to integrate into studying HIV prevention interventions, in addition to the inclusion of separate drug splitting indicators into future research studies.

We also found that women and syringe service utilizers were more likely to have participated in the dyad session. Patterns of self-selecting into a dyad session are essential for understanding how to design broadly oriented peer-based HIV prevention interventions. Although most analyses on couples-based interventions have randomized sex participation equally, there is evidence that women may self-select due to previous research demonstrating that women are at heightened risk for HIV/HCV in injecting partnerships.^5,10,22–25^ Although we cannot delineate whether women were more motivated to participate in our dyad intervention as a function of their desire to reduce risk with partners from other motivators, we know that injection risk decision making can be influenced by the relationship dynamics between partners, especially in light of sex imbalances, partner pressure, and intimate partner violence.^7,8,9,26^ In addition, it is possible that those who chose to participate in the dyadic session were already accessing harm reduction services and were more motivated to learn and practice safer harm reduction behaviors than those who did not participate. Future research is necessary to understand the effects by sex and the interpersonal dynamics that influence dyadic interventions on reducing risk among people who inject drugs.

### Limitations

The study has limitations to note. The reduction in sharing equipment by level of dyad participation may be subjected to social desirability bias by sheer reporting of risk-taking behaviors. For example, participants completing an HIV risk reduction program may feel expected to report risk-reducing behaviors. The underreporting of risk by dyad participants may artificially inflate the effect of the intervention. However, it is impossible to understand the degree in underreporting among both groups and the direction of a biased effect. Therefore, it is assumed that both groups report responses equally. In attempt to control for this bias, self-administration of an audio-recorded-assisted survey was conducted in a confidential setting. The sample is also prone to study selection bias as the dyad participants were not randomized into the dyad session and were self-selected. Although the multivariable model adjusted for sex differences to control for the sex selection bias into opting into dyad participation, other factors related to behavioral readiness for syringe sharing were excluded from the model. For example, dyad participants were previously engaged in needle exchange services more than nondyad participants, thus influencing behavioral change readiness and postintervention equipment sharing. However, we found that the inclusion of needle exchange services did not change the effect when considering for model inclusion. In addition, the dyad’s reduced sharing behavior across the dyad group may be attributed to the 7-session peer-based behavioral intervention previously reported by Tobin et al^16^ that effectively reduced HIV risk-taking behaviors among intervention participants compared with control, therefore minimizing the sole influence of the dyad session on behavioral change. Furthermore, a small sample size may dilute a statistical association between the dyad and nondyad groups and limits the internal validity of the findings. Previous intimate couples-based interventions also employed small sample sizes and did not capture a model effect.^5,15^ However, taken together, the findings in light of previous research are suggestive that the dyadic component may have an influence in a larger scale intervention.

Given the shared nature of injection risk, particularly as clustered within social network relationships, dyadic behavior change interventions may offer a potential mechanism for shared risk reduction, although this study did not capture an effect. It would be worthwhile for future studies to explore the effects of dyadic interventions on the risk behaviors of both injection partners and specifically behaviors within that relationship.
